# How Distance Affects Semantic Integration in Discourse: Evidence from Event-Related Potentials

**DOI:** 10.1371/journal.pone.0142967

**Published:** 2015-11-16

**Authors:** Xiaohong Yang, Shuang Chen, Xuhai Chen, Yufang Yang

**Affiliations:** 1 Key Laboratory of Behavioral Science, Institute of Psychology, Chinese Academy of Sciences, Beijing, China; 2 Jiangsu Collaborative Innovation Center for Language Ability, Jiangsu Normal University, Xuzhou, China; 3 Department of Psychology, Zhejiang Normal University, Jinhua, China; 4 School of Psychology, Shannxi Normal University, Xi’an, China; The University of Nottingham, UNITED KINGDOM

## Abstract

Event-related potentials were used to investigate whether semantic integration in discourse is influenced by the number of intervening sentences between the endpoints of integration. Readers read discourses in which the last sentence contained a critical word that was either congruent or incongruent with the information introduced in the first sentence. Furthermore, for the short discourses, the first and last sentence were intervened by only one sentence while for the long discourses, they were intervened by three sentences. We found that the incongruent words elicited an N400 effect for both the short and long discourses. However, a P600 effect was only observed for the long discourses, but not for the short ones. These results suggest that although readers can successfully integrate upcoming words into the existing discourse representation, the effort required for this integration process is modulated by the number of intervening sentences. Thus, discourse distance as measured by the number of intervening sentences should be taken as an important factor for semantic integration in discourse.

## Introduction

One of the central research topics in psycholinguistics is understanding the nature of discourse comprehension. In general, establishing any kind of discourse representation requires an integration process that brings an upcoming new item into contact with its prior mental representation of the discourse. However, this task is not trivial. The elements to be integrated need not be close together, but may be separated by several sentences in the discourse. This gives rise to the following theoretical question: whether semantic integration is influenced by global factors such as the number of intervening sentences in a discourse. The present study explores the unique contribution of discourse distance to online semantic integration in discourse processing.

Processing any discourse requires integrating each upcoming word with its prior discourse context. There have been multiple demonstrations that global discourse context can override early semantic interpretations in local sentence context[1–14]. The ERP component that is most consistently reported for semantic integration is the N400, a negative-going wave that peaks at approximately 400ms post stimulus onset. Generally, as the degree of semantic fit between a word and its context increases, the amplitude of the N400 goes down[15]. Thus, the N400 has been assumed to be associated with the ease with which the meaning of a word is accessed and integrated into the preceding context. Previous studies have consistently found that semantically incongruent words elicit larger N400s than semantically congruent words, which is referred to as the N400 effect[16]. For example, by placing sentences such as “*Jane told her brother that he was exceptionally quick/ slow*” in a discourse that rendered one of the critical words anomalous (e.g. *Jane’s brother had in fact done something very quickly*), Van Berkum and colleagues[7,8]found a larger N400 for the discourse-dependent incongruent words (e.g. *slow*) than the congruent words (e.g. *quick*). This suggests that congruency with the discourse context immediately influences lexical-semantic processing of the critical words, even when these words are congruent in the local sentence context.

Besides the N400 effect, the P600 effect is also reported for semantic integration in discourse context[17,18]. The P600 is a positive-going wave that starts at about 500ms post stimulus onset and reaches its maximum at about 600 ms. In some studies, anomalies having a good fit to the global discourse context did not induce an N400 effect, but a P600 effect instead. For instance, Sanford et al.[17,18] found that difficult anomalies having a good fit to general context (e.g., the *[victim]* in “a 10-year sentence was given to the *[victim]”* in a situational scenario about *child abuse*) did not produce an N400 effect, whereas control “easy-to-detect” anomalies did (e.g., the *[*letters*]* in “to think of new ways to sell more *[*letters*]”* in a situational scenario about *record shop*). This suggests that a good fit to global context serves to reduce the extent of local semantic processing so that an anomaly is not detected. Similarly, instead of an N400 effect, Nieuwland and Van Berkum[17] reported a P600 effect when listeners detected anomalies (e.g., ‘‘the woman told the *[suitcase]*” in a scenario in which the suitcase was a very salient part of the setting). This again suggests that words with a good fit to global scenario only receive shallow semantic processing, thus providing further support for a strong and immediate effect of discourse context.

It is worthy to note that the P600 component was originally considered to be an index of syntactic processing difficulties such as the processing of syntactic violation or non-preferred syntactic structure[19–21]. However, the fact that the P600 is also elicited by grammatically and syntactically well-formed structures indicates that it is not a simple indicator of syntactic processing. The functional significance of the P600 to semantic anomalies has thus triggered considerable debate in the literature[22]. It has been proposed that the P600 may reflect the brain’s natural electrophysiological reflection of integration cost[23,24], a conflict monitoring process [25], or a more general integration process involving various information such as semantics, pragmatics, prosody, and others[26–28]. Recently, following a growing number of studies, Brouwer and colleagues[29] have proposed the Retrieval-Integration account which interprets the N400 amplitude as reflecting a memory-retrieval phase in which all the information linked to a upcoming word is retrieved and the P600 amplitude as reflecting mental representation construction.

The N400 and P600 effect that were reported for discourse-dependent anomalies have thus provided evidence that each upcoming word in a discourse is integrated into its existing discourse representation during online discourse comprehension. However, during discourse processing, it frequently happens that an upcoming word needs to be interpreted with information that came much earlier in the discourse. Therefore, the question that arises is how distance affects semantic integration during discourse comprehension.

The effect of distance is important and relevant to the development of discourse comprehension theories. Major theories of discourse comprehension, such as the Construction-Integration model[30], the resonance model[31], and the landscape model[32], all assert that distance could be a critical factor affecting the construction of discourse representation. These theories have outlined two essential processes during reading: memory-based activation and constructionist processes. According to these theories, distance affects the memory-based activation processes of discourse comprehension: Increasing the distance between temporally distal parts of the discourse degrades the underlying memory processes between these two parts.

Ample evidences for distance effects have been found, mainly coming from behavioral studies. For instance, in the case of anaphor resolution, it has been showed that the time to process an anaphor depends on distance: Antecedents that are more physically distant require more time to be resolved [33,34]. Also, when a bridging inference between a category in one sentence and a exemplar in the following sentence is required, reading times on the target line are shorter when the previous mention is near than when the previous mention is more distant[35]. Crucially, it is interesting to note that in the existing literature, the general consensus is that distance affects the memory-based processes. However, whether distance affects the effortful constructionist processes is far from settled.

Despite the importance attached to distance effects, however, evidence for on line distance effects is quite narrow methodologically: It is restricted almost exclusively to behavioral measures. Given the significance of distance effects to theory development, we believe that it is important to investigate its cognitive neural mechanisms and broaden its empirical support to neural measures. Thus, our first aim is to extend the empirical support of distance effects to include ERP measures. Second, with sensitive brain components like N400 and P600 indexing early and late stage of integration respectively [5,29], we aim to further delineate the effect of distance on different stages of discourse integration processes.

Here, we present an ERP experiment that seeks to address how distance affects semantic integration during discourse comprehension. Two kinds of discourses were tested: short discourses consisting of three sentences and long discourses consisting of five sentences. We manipulated semantic integration in such a way that the final sentence of each discourse contained a critical word that was either congruent (e.g., *sadnes*s in the example of Table 1) or incongruent (e.g., *happines*s) only when interpreted with the information introduced in the first sentence (e.g., *got fired*). In this way, in the short discourses, semantic integration involves only one intervening sentence between the heads of the two projections being integrated together while in the long discourses, semantic integration involves three intervening sentences. Crucially, the intervening sentences in both conditions continue the first sentence in a coherent way (e.g. "*He was sorting files in his office*," in the short condition and " *He was sorting files in his office*, *he seemed very busy*, *not even have time to talk*," in the long condition), but do not elaborate on the target point that is to be integrated in the first sentence (e.g. *got fired*). Thus, the structure for the short condition is "A, then B, then E (which is to be integrated with A)", while the structure for the long condition is "A, then B + C + D, then E (which is to be integrated with A)". This manipulation allows us to test directly the influence of distance on semantic integration in online discourse processing. We also matched the number of discourse referents introduced in the two conditions so that the influence of discourse distance would not be confounded by the number of discourse referents.

**Table 1:** Example stimuli used in the present study.

**Lead-in sentence:**
老王刚刚被公司解雇了,
Old Wang just got fired from the company,
**Short distance condition:**
他正在办公室整理文件,
He was sorting files in his office,
**Long distance condition:**
他正在办公室整理文件, 看上去非常忙碌, 连说话的时间都没有,
He was sorting files in his office, he seemed very busy, not even have time to talk,
**Target sentence:**
仔细看去老王的脸上满是**落寞 (欢快)** 的神情。
A closer look revealed that his face was full of **sadness (happiness)**.

It has been argued that the dependent variables in the inconsistency paradigm actually reflect the processes when readers recognized the inconsistency and engaged in some sort of inference process to try to reestablish coherence[32]. In the present study, the farthest distance was such that, according to ERP results from previous studies[5,36], we were reasonably sure that the anomalies could be successfully detected and the N400 effect would be elicited for the incongruous words in both short and long discourses. If after readers recognized the anomalies, they engaged in some sort of reconstruction processes to establish coherence, we can expect that the P600 effect should be more pronounced in the long condition because the discourses in this condition are longer and more cognitive effort is required to reconstruct a longer discourse.

## Method

### 2.1 Ethics Statement

All participants provided written informed consent in accordance with the Declaration of Helsinki. The ethics committee of the Institute of Psychology, Chinese Academy of Sciences approved this study, its participant-recruitment procedure and its methodology.

### 2.2 Participants

Thirty-six university students (18 males; mean age = 23.3 years; SD = 3.1 years) served as paid volunteers. All were native speakers of Chinese and were right-handed with normal or corrected-to-normal vision. The data of eight subjects (five males) were excluded due to excessive EEG-artifacts or technical problems with recording. Thus, twenty-eight participants remained for subsequent analysis.

### 2.3 Materials

120 sets of discourses were used in the present study. For each discourse, the last sentence contained a critical word which was either congruent or incongruent with the relevant information in the first sentence. The number of intervening sentences between the first and the last sentence was one and three in the short and long condition respectively. The incongruent and congruent words were matched in average frequency per million (incongruent: *M* = 190.00, *SD* = 293.60; congruent: *M* = 178.55, *SD* = 323.30; *t*_(119)_ = 0.27, *p* > .1) on the basis of information provided by the Chinese Linguistic Data Consortium[37]. The 120 sets of discourses were arranged according to a Latin Square design into four lists so that each discourse set was presented only once within each list. Each list contained 30 discourses per condition. To each list 100 filler discourses were also added.

In order to match the cloze probability of the critical words across the experimental conditions, we truncated the discourses before the critical words and asked 22 subjects to write down the first word that came to mind to complete the discourses. The stimuli were assigned into two lists. In each list, participants could only see the short condition or the long condition of the same discourse set and there were an equal number of discourses from the two conditions. Results showed that the cloze probability for the incongruent words was equally zero for both the short and long condition. The cloze probability for the congruent words was 31.91% (SD = 16.10%) for the short condition and 30.82% (SD = 16.67%) for the long condition. A t-test with distance (2 levels: short vs. long) as independent variable and cloze probability of the congruent words as dependent variable was thus conducted. No significant difference was found between the two conditions (*t*_(21)_ = 0.34, *p* >. 1).

To assess the degree of semantic acceptability across conditions, we asked another 16 subjects to rate the acceptability of the discourses on a 5-point Likert scale (5 indicates the most acceptable). The participants were assigned one of the four counterbalanced lists. A Repeated-measures ANOVA was conducted with distance (2 levels: short vs. long) and congruence (2 levels: congruent vs. incongruent) as independent variables and acceptability ratings as dependent variable. The results revealed a main effect of congruence, *F* (1, 15) = 20.42, *p* < .001, *η* ^*2*^_*partial*_ = .58 (mean ± SD = 4.38 ± 1.62; 2.42 ± 1.15; 4.17 ± 1.38; and 2.26 ± 0.79 for short/congruent, short/incongruent, long/congruent, and long/incongruent conditions respectively), while no significant main effect of distance or significant interaction was found (*Fs* < 2.20, *ps* > .1).

### 2.4 Procedure

Participants were seated in a comfortable chair approximately 60 cm in front of a monitor in a sound-attenuating shielded chamber. The discourses were presented in white color on a black background. A trial began with a fixation cross which remained on the screen for 1000 ms. Then the first two sentences (for the short condition) or the first four sentences (for the long condition) of the discourses appeared on the screen sentence by sentence. Participants were told to press a button when they finished reading the sentences. The last sentence was presented one word at a time. Each word was presented for 400 ms, with an inter-stimulus interval of 200 ms. After 1/3 of the trials they were asked to respond to a true/false comprehension question by pressing one of the two appropriately marked keys. Half of the questions required a “True” response and half a “False” response (e.g., *Was old Wang fired from the company*?). After a short practice of 8 trials, the materials were presented in four blocks, with each block lasting about 10 min, separated by brief resting periods. The participants were free to move or blink during the presentation of the sentences but were instructed not to move or blink during the presentation of the words on the computer screen.

### 2.5 EEG Recording and analysis

EEG was recorded with 64 Ag/AgCl electrodes mounted in an elastic cap according to the international 10–20 system (Neuroscan Inc.). The EEG was sampled at 500 Hz with an amplifier bandpass of 0.01–100 Hz. EEG data were referenced online to the right mastoid and re-referenced offline to the algebraic average of both mastoids. Vertical EOG was recorded by electrodes placed above and below the left eye. A right-to-left canthal bipolar montage was used to monitor horizontal eye movements. All electrode impedances were kept below 5 kΩ.

Scan 4.3 software (Neuroscan Labs, TEXAS, USA) was used to preprocess the EEG data. EEG and EOG records were screened for eye movements and electrode drifting. The data were filtered off-line with a 30 Hz low-pass filter. Then the data were segmented from 200 ms before to 1000 ms after the onset of the critical words, with baseline correction from 200 to 0 ms preceding word onset. After that, an artifact rejection criterion of ± 75 μV was applied. Overall, 12% contaminated trials were rejected, with rejections being equally distributed across the four conditions. There were 26.17 ± 2.91, 26.14 ± 3.18, 26.54 ± 2.55, and 26.43 ± 2.62 artifact-free trials obtained for the short/congruent, short/incongruent, long/congruent, and long/incongruent conditions respectively.

**Fig 1:**
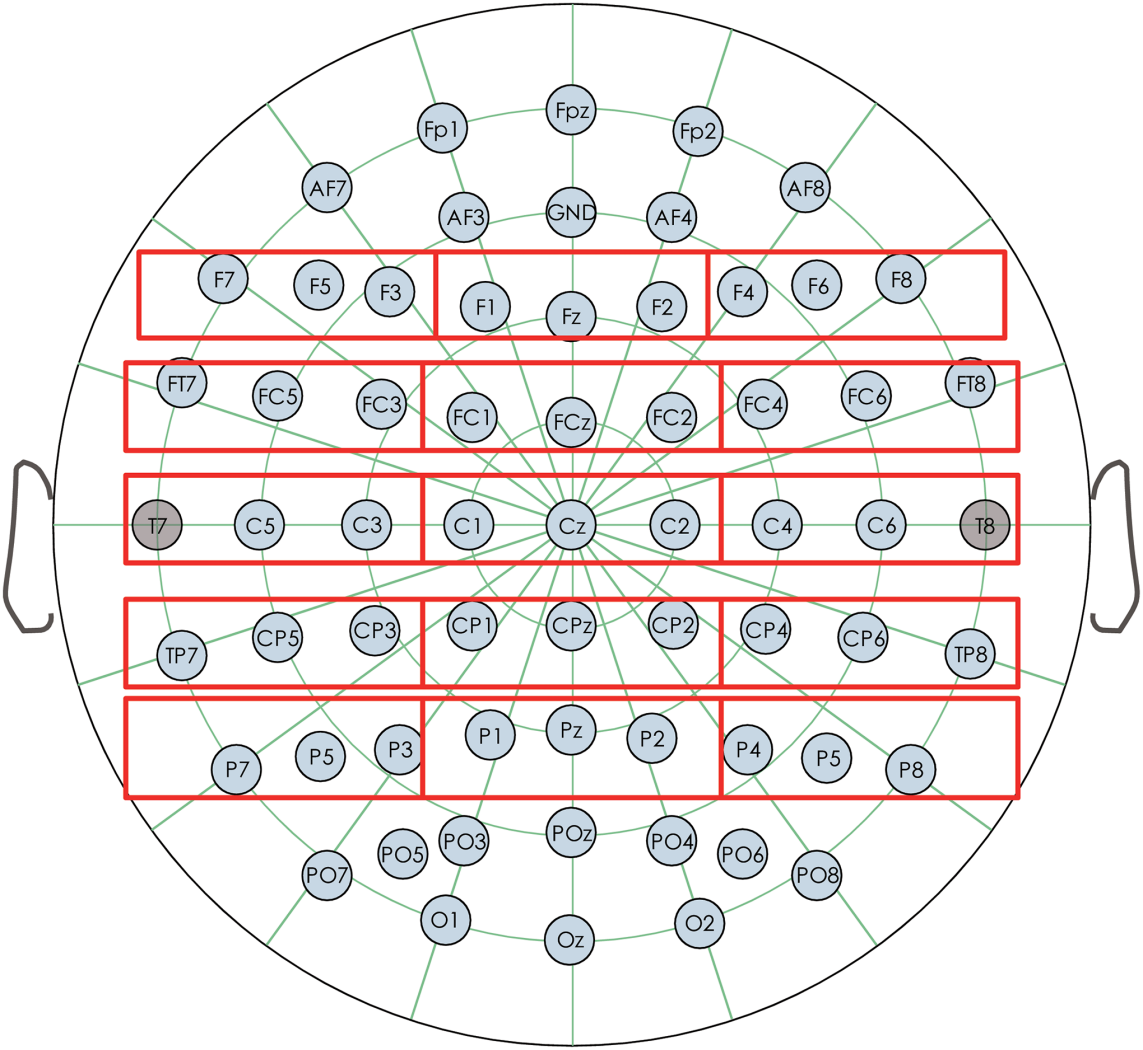
The electrodes used in the present study.

Average waveforms were computed across all trials per condition for the 28 participants within each of the selected time windows (see the Results section). Analyses of variance (ANOVAs) were conducted with distance (2 levels: short vs. long) and congruence (2 levels: congruent vs. incongruent) as independent variables. Two topographical factors were also included as independent variables in the ANOVAs in order to cover distributional differences in both the anterior–posterior and the left–right dimensions. The first was laterality, which had three levels: left, medial, and right[[38,39]. The second was ant–pos, which included five levels: frontal, frontal-central, central, central-parietal, and parietal[39,40]. In this way, we created 15 regions of interests (ROI) out of 43 electrodes, each containing 3 or 2 electrodes (as shown in Fig 1): left frontal (F3, F5, F7), left frontal-central (FC3, FC5, FT7), left central (C3, C5), left central-parietal (CP3, CP5, TP7), left parietal (P3, P5, P7), medial frontal (F1, FZ, F2), medial frontal-central (FC1, FCZ, FC2), medial central (C1, CZ, C2), medial central-parietal (CP1, CPZ, CP2), medial parietal (P1, PZ, P2), right frontal (F4, F6, F8), right frontal-central (FC4, FC6, FT8), right central (C4, C6), right central-parietal (CP4, CP6, TP8), and right parietal (P4, P6, P8). The dependent variables were the N400 and P600 amplitude. When the degree of freedom in the numerator was larger than one, the Greenhouse–Geisser correction was applied. The reported p-values were based on the Greenhouse ± Geisser corrections. However, the uncorrected degrees of freedom were reported.

## Results

### 3.1 Behavioral measures

On average, participants gave a correct response of 80% to the comprehension questions, indicating that they were reading the discourses attentively. A two-factor repeated-measures ANOVA was conducted with distance (2 levels: short vs. long) and congruence (2 levels: congruent vs. incongruent) as independent variables and accuracy rates as dependent variable. The results revealed neither the main effect of discourse distance or congruence, nor the interaction between the two factors (mean ± SD = 0.82 ± 0.13; 0.84 ± 0.12; 0.86 ± 0.13; and 0.83 ± 0.11 for short/congruent, short/incongruent, long/congruent, and long/incongruent conditions respectively, *Fs < 1*, *ps >*.*1)*.

### 3.2 ERP results

**Fig 2:**
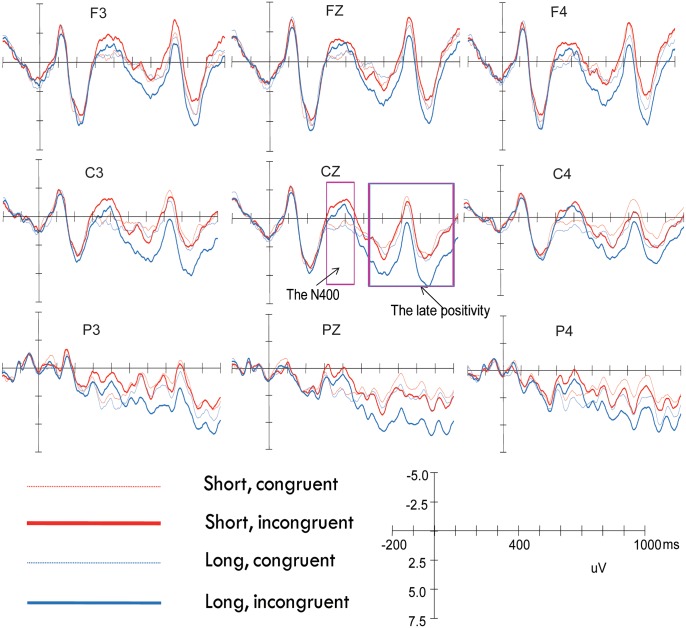
Grand average waveforms elicited by the four conditions at nine selected electrode sites. Waveforms are time-locked to the onset of the critical words and negative amplitude is plotted up.

**Fig 3:**
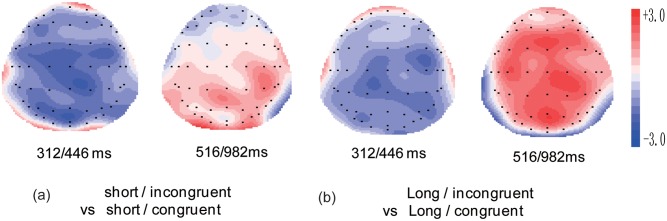
Topographies of the difference wave formed by subtracting ERPs to the short/ congruent from short /incongruent (a), and ERPs to the long/ congruent from long /incongruent (b) in selected time periods.

The grand average waveforms elicited by the four conditions at nine representative electrodes are presented in Fig 2 and the topographies showing the average voltage differences for the different contrasts are presented in Fig 3. As shown in Figs 2 and 3, relative to the congruent words, the incongruent words elicited both an N400 effect and a P600 effect. To determine the precise onset and offset of the N400 and P600 component, the method proposed by Guthrie and Buchwald[41]was used. To compute the ranges of significant differences between two ERPs, this method uses adjacent tests as replications, requiring a temporal region of consecutive tests to be significant for any one test to be judged as significant at *p* < .05 after accounting for the temporal autocorrelation of the waveform[42]. This procedure controls for Type I error rates without using conservative multiple comparison methods and has been applied widely to ERP data in the recent past[43–45]. Application of the Guthrie and Buchwald[41] method revealed significant differences during the epoch of 312–446 and 516–982, which were determined as the time window for the N400 and P600 respectively.

#### The N400 Time window (312-446ms)

The statistical analysis of the N400 component revealed a significant main effect of congruence [F (1, 27) = 24.39, *p* < .001, *η* ^2^_partial_ = .48]. Mean amplitude was more negative going for the incongruent words than the congruent words, suggesting that an N400 effect was obtained. There was an interaction between congruence and ant–pos [F (4, 108) = 2.50, *p* < .05, *η* ^2^_partial_ = .08]. Simple effect analysis, revealed that the N400 effect was obtained over all the five ant-pos levels, but most prominent over central-posterior areas: frontal[F (1, 27) = 9.38, *p* < .01, *η* ^2^_partial_ = .26], frontal-central[F (1, 27) = 14.56, *p* < .01, *η* ^2^_partial_ = .35], central[F (1, 27) = 20.91, *p* < .001, *η* ^2^_partial_ = .44], central-parietal[F (1, 27) = 22.96, *p* < .001, *η* ^2^_partial_ = .46], and parietal[F (1, 27) = 30.59, *p* < .001, *η* ^2^_partial_ = .53]. There was also a significant main effect of distance [F (1, 27) = 5.13, *p* < .05, *η* ^2^_partial_ = .16], with mean amplitude more negative going for the short condition than for the long condition. No significant interactions involving congruence and distance were found. Therefore, the long and the short conditions did not differ from each other as far as the N400 effect is concerned.

#### The P600 (516-982ms)

The overall results revealed a significant main effect of congruence [F (1, 27) = 13.56, *p* < .01, *η* ^2^_partial_ = .33], with incongruent words eliciting a larger positivity than congruent words. This indicates that the P600 effect was obtained. There was also a significant congruence by ant–pos interaction [F (4, 108) = 8.23, *p* < .001, *η* ^2^_partial_ = .24]. Simple effect analysis revealed that the P600 effect was obtained over frontal-central[F (1, 27) = 7.51, *p* < .05, *η* ^2^_partial_ = .22], central[F (1, 27) = 15.38, *p* < .01, *η* ^2^_partial_ = .36], central-parietal[F (1, 27) = 22.60, *p* < .001, *η* ^2^_partial_ = .46], and parietal areas[F (1, 27) = 15.55, *p* < .01, *η* ^2^_partial_ = .37], but not frontal areas[F (1, 27) = 0.32, *p* >.1, *η* ^2^_partial_ = .01]. Thus, the P600 effect was most prominent over central- parietal areas.

More importantly, the overall results also revealed a marginally significant distance × congruence interaction [F (1, 27) = 3.53, *p* = .07, *η* ^2^_partial_ = .12] and a significant distance × congruence × hemisphere interaction [F (2, 54) = 5.03, *p* < .05, *η* ^2^_partial_ = .16]. To resolve the distance × congruence × hemisphere interaction, we broke down this interaction by doing separate analysis for each distance condition. For the short condition, an ANOVA with congruence and hemisphere as two within-subject factors revealed nether a significant main effect of congruence nor any interaction between congruence and hemisphere [Fs < 2.13, *p* >.1], suggesting that no P600 effect was found for the short condition. For the long condition, an ANOVA with congruence and hemisphere as two within-subject factors revealed a significant main effect of congruence [F (1, 27) = 10.15, *p* < .01, *η* ^2^_partial_ = .27] and a significant interaction between congruence and hemisphere [F (2, 54) = 9.05, *p* < .01, *η* ^2^_partial_ = .25]. Simple effect analysis, however, showed that incongruous words elicited a larger positivity in all the three hemispheres [left: F (1, 27) = 6.04, *p* < .05, *η* ^2^_partial_ = .18; medial: F (1, 27) = 15.45, *p* < .01, *η* ^2^_partial_ = .36; right: F (1, 27) = 5.66, *p* < .05, *η* ^2^_partial_ = .17]. Taken together, a P600 effect was only observed for the long discourses, but not for the short ones.

## Discussions

The goal of the experiment was to investigate the effect of distance on semantic integration in discourse context. To this aim, discourses were tested in which the last sentence contained a critical word that was either congruent or incongruent with the information presented in the first sentence. In addition, the first sentence was separated from the last sentence either by one sentence or three sentences. We found that an N400 effect was elicited for both the short and long discourses. However, a P600 effect was only observed for the long discourses, but not for the short ones. These results suggest that distance does not affect the early stage in which information is accessed and integrated into its preceding context, but affects the later stage in which readers initiate a reconstruction process to establish global coherence.

### 4.1 Effect of semantic congruence

In traditional theories about meaning interpretation, the dominant view is that when understanding an utterance, local sentence constraints (lexical-semantic) are handled at an early stage whereas others (contextual constraints) can only be brought to bear at an later stage [30,46,47]. In the current study, anomalies that were fully congruent in local sentence context but were only incongruent with respect to the prior discourse elicited an N400 effect. This N400 effect was widely distributed across the scalp, but was largest over central and posterior sites, paralleling the topographical distribution of the classic N400 effect[16]. The presence of the N400 effect elicited by discourse-dependent anomalies is consistent with previous studies that found an immediate effect of discourse context on the processing of semantic anomalies[1,5,7,8]. This result is fully consistent with the notion that there is no initial stage during which lexical processing of an upcoming word is computed with respect to local sentence context, independent of the discourse context in which the sentence occurs. As such, our results thus strongly support the notion that discourse interpretation involves the immediate mapping of incoming word meanings onto the widest discourse context available[15].

Interestingly, the N400 effect was followed by the P600 effect. This effect was obtained from frontal-central to parietal areas, but was most prominently observed over central-parietal electrodes, resembling the scalp distribution of the semantic P600 reported in previous studies[18,28]. The finding of the P600 effect is consistent with a number of studies that report a P600 effect for semantically anomalous words in discourse processing[17,18]. Although a N400 + P600 complex has been observed for sentence processing for many years, the functional interpretation of the post-N400 positivity has been subject to a substantial amount of debate in the literature (for a review, please see Van Petten and Luka[22]). Given that the P600 effect was only observed for the long discourses in the present study, we speculate that it reflects an enhanced cost of processing a larger discourse context when readers initiate a reinterpretation process to establish global coherence[29].

### 4.2 Effect of distance manipulation

Interestingly, our manipulation of distance did not produce any difference in the N400 time window. According to our ERP results, for both the short and long discourses, readers started to integrate local lexical information with prior discourse context in the N400 time window. Thus, our data provide no indication whatsoever that the language comprehension system is slower in relating a new word to the prior discourse when there is more intervening material than when there is less intervening material. In the present study, semantic integration in the short discourses involved only one intervening sentence between the heads of the two projections being integrated together while in the long discourses, semantic integration involved three intervening sentences. According to the ERP results from a related study[36], anomalies can be successfully detected even when three long sentences intervened between the anomalies and the earlier to-be-integrated information. Thus, the absence of an N400 difference between the short and long conditions may result from the fact in both conditions, earlier information in the discourse can be easily reactivated and readers can relate the discourse-dependent anomalies in the final sentence to the target information introduced in the first sentence with ease.

The absence of the N400 difference between the short and the long conditions can also be due to the fact that the anomalies were equally unexpected in both distance conditions. Numerous studies have demonstrated that the amplitude of N400 is highly correlated with cloze probability: N400s to the equally low cloze probability completions of strongly constraining sentences were statistically indistinguishable (see Kutas and Federmeier[16] for a review). As mentioned in Section 2.3, our pretests of experimental material have revealed that the cloze probability for the incongruent words was equally zero for both the short and long condition. Previous findings have showed that poor-fit words require full processing, which, in general, elicits a classic N400 effect[18]. Thus, given that the incongruous target words were equally unexpected and having a poor fit to global discourse context in both conditions, it is reasonable that they were fully processed and elicited full N400 effects in both conditions.

Recent studies have argued that many reading processes are incomplete and that readers settle for a "good enough interpretation" if this underspecification does not disturb the comprehension process[18,48]. In the studies using ERPs, this underspecification often results in the absence of the N400 effect for anomalous words, which was referred to as "semantic illusion" [9,17,18]. The fact that in the present study, full N400 effects were found for both distance conditions suggests that adding additional information between the heads of the two projections being integrated together did not necessarily lead to semantic illusion.

We found similar N400 effects for incongruent compared to congruent sentences for both distances. Thus, the initial process of relating upcoming words to prior discourse context seems to be independent of working memory effect. Previous studies have yielded divergent results concerning the role of working memory on the N400 effect. Significant effects of working memory on the N400 have been observed in several studies[49–51]. For instance, Hammer and colleagues[49] found that increased working memory load reduced the N400 effect. Similar effects have also been observed in the study by D'Arcy and colleagues[50], which showed that increased working memory load in a semantic N400 task not only reduced the N400 effect, but also resulted in delayed N400 amplitudes as compared to a lower working memory load. However, there are also studies which suggested that the N400 is independent of working memory differences[52–54]. For instance, Van Schie et al[54] reported that visual working memory load did not modulate the N400 response, but modulated the late positivity instead. Our results are consistent with those of van Schie et al[54] and provide further evidence that working memory differences may not necessarily produce an effect on the N400, but may influence the late positivity instead.

According to the resonance model[31], distance affects the memory-based processes either because resonance is a function of the strength of a concept and strength decays over time, or because more recently presented concepts are more likely to overlap with the current contents of working memory. In the present study, our materials were constructed in such a way that concept overlap was equated. Thus, if there were any distance effects, it would be the result of activation decaying over time. However, we found that the N400 effect was not affected by distance manipulation. It is well known that N400 amplitude can be taken to reflect the retrieval of the meaning of a word from long-term memory[16,29]. Therefore, the absence of a N400 difference between the two distance conditions stands in sharp contrast to the memory-based account of discourse processing. We cannot entirely rule out the possibility that activation decayed over time in the present study. However, based on the current results, we can reasonably conclude that the addition of two more intervening sentences in the long discourse did not significantly affect the memory-based process. Thus, our results indicate that when a discourse representation is built, it is not quickly lost from memory.

Crucially, our manipulation of distance was found to affect the P600 effect. The P600 effect was found for the long discourses, but not for the short ones. This result compares favorably with previous findings that reported the presence of a P600 effect when additional processing (as compared to the control condition) was required in order to arrive at a coherent mental representation([23,24], and see Brouwer, Fitz, and Hoeks [29] for a review). It has been suggested that the P600 effect may reflect the effort in reworking an initial mental representation[29]. Thus, the presence of the P600 effect for the long discourses may be due to the fact that reader initiated a reconstruction process to establish global coherence and the reconstruction of a longer discourse could be associated with a higher cost because more substantial reconstruction is needed to incorporate the current linguistic input into the larger context. The combined presence of the N400 and P600 effect to the long discourses may also suggest that although readers could successfully integrate the discourse-dependent-anomalies to the wider discourse context in the long condition, more computational effort is needed.

In discourse processing literature, memory-based and constructionist processes have been described as two most essential components during reading by major theories of discourse comprehension[30–32]. Passive, autonomous activation of information in memory is followed by an active, constructionist building process. There is ample evidence showing that memory-based and constructionist processes interact during discourse comprehension[55–56]. To establish coherence, readers must activate the to-be-connected information and determine what, if any, connections exist between the activated pieces of information. In other words, memory-based processes provide the input to the constructionist processes, and the product from the constructionist processes determines whether the memory-based input is sufficient for comprehension[32]. Previous studies have repeatedly demonstrated that distance has significant effects on the memory-based processes (see Myers and O'Brien[31] for a review). However, the results of the present study revealed that the initial memory-based processes were not affected by distance, but subsequent constructionist processes were affected by distance. It should be noted that similar findings have also been observed in the literature, showing that distance alone does not necessarily produce an effect on memory-based processes[57,58]. Thus, the present study extends findings from previous literature by showing that distance can lead to an increase effort in the constructionist processes even when the memory-based processes of activation are not significantly affected.

In conclusion, our results suggest that although readers can successfully integrate upcoming words into the existing discourse representation, the effort required for this integration process is modulated by the number of intervening sentences. Our findings have major implications for how distance bears on theory development: Distance was found to significantly affect the effortful constructionist processes, but not the memory-based processes. We are aware that the present data do not provide conclusive evidence concerning the effect of distance on semantic integration. We believe that graded manipulations of distance will shed further light on this issue. We also caution that individual variations in working memory may be an important factor underlying long-distance interpretations and future research is needed to fully understand the relationship between working memory capacity and the effects of distance in discourse processing. Finally, in the current study, the anomalies were all easy-to-detect anomalies. Future research is needed to compare the effects of distance on both easy-to-detect anomalies and difficult-to-detect ones so as to further elucidate the effects of distance on memory-based activation processes.
